# Notes and Notices

**Published:** 1898-11

**Authors:** 


### NOTES AND NOTICES.

DR. B. F. BETTS, 1609 Girard Avenue, Philadelphia, having entirely recovered from recent illness, has resumed practice as heretofore: September 15th, 1898.

DR. GUY E. MANNING has removed his offices to Spring Valley Building, over "City of Paris," corner of Geary and Stockton Streets, rooms 106 and 108, San Francisco, Cal. Office hours, 3 to 5 P. M.; Sundays, 11 to 12 A. M. Telephone Black 286.

THE RED CROSS SOCIETY.—The Associate Society of the Red Cross of Philadelphia is maintaining its hospital at Camp Meade. Patients too ill to be removed to the city hospitals are taken there. Its capacity to receive all these cases is inadequate because of the Society's inability to secure experienced women nurses for the nominal sum of sixteen dollars per month. Nurses willing to make the sacrifice and to engage in this laudable work will kindly apply by letter or report to "The Committee on Nurses," 1501 Chestnut Street, Philadelphia. Funds are needed to meet the increasing demands made upon the Society.—*Executive Committee.*

SEE the girls in another column carrying large baskets of grapes to a winery in Portugal for making wine. It is worth reading about. Speer of New Jersey makes wine from the same grape. His wines are unsurpassed by any in the world.

TREATMENT OF BURNS.—Keen and DaCosta (*Practitioner*) claim excellent results from the following use of normal salt solution (one teasponful table salt to the pint of water): The injured part is wrapped in absorbent cotton, soaked with normal salt solution, and a bandage applied which contains a trap door for the frequent addition of the solution, and the part put strictly at rest. They claim that pain is quickly allayed and that granulation is very rapid, while if skin grafts are required the raw surface is prepared. Pozzi uses saturated solution of Potassium-nitrate in the same way and claims rapid abolishment of pain. Haas gives the following recipe:

℞ Aristol, . . . . . . . . . . . . . . . . . . grs. 75 to 150
Olive oil, . . . . . . . . . . . . . . . . . . " 300
Lanolin.
Vaselin, . . . . . . . . . . . . . . . . . . aa " 600

M. Sig.—Use as external application.—*Buffalo Medical Journal, April, 1898.*

APPENDICITIS AND MONEY.—Young doctor: "Did you diagnose his case as appendicitis, or merely the cramps?" Old doctor: "Cramps. He didn't have money enough for appendicitis."—*Life.*

VALUABLE IN CHANGEABLE WEATHER.—People are benefited by the use of Speer's Port Grape Wine, especially ladies. It purifies the blood and makes their eyes shine like stars.

POEM BY THE EDITOR.—The other day while in "our den," these curious lines came from our pen, as we read o'er and o'er again the Poem of the Editor. We thought while writing, 'twould be right to place before the reader's sight these lines, and let him judge aright the Poem of the Editor. Here it is:

THE EDITOR—HIS POEM.

Who weeps with you when you are sad, and laughs with you when you are glad, and swears with you when you are mad? The Editor. Who has to be both kind and wise, and never (hardly ever) lies, and when he does creates surprise? The Editor. Who owns a heart as well as cheek, is possessed of spirit, proud but meek, and lives on forty cents a week?—*Weekly Enterprise, Hamilton, Va.*

To the above THE HOMŒOPATHIC PHYSICIAN adds:

Who waits in patience day by day, and longeth for subscribers' pay, and listens to the printer's lay? The Editor.

FEMALES and weakly persons at this season should use Speer's Port Grape Wine. Physicians recommend it as a strengthening and blood-purifying tonic, and the best wine to be obtained.

THACKERAY'S DAUGHTER AND HOMŒOPATHY.—There is an interesting passage in Mrs. Ritchie's "History of Pendennis,"quoted in the *Daily Chronicle Review*, May 17th, which shows a wholesome appreciation of Homœopathy in the Thackeray household:

"When Helen Pendennis, so to speak, lay dying, Thackeray's younger daughter said, 'Oh, papa, do make her well again; she can have a regular doctor and be almost dead, and then will come a homœopathic doctor, who will make her well, you know." "Pendennis" was dedicated to Dr. Elliotson, who lost his practice through his courage in investigating the phenomena of hypnotism, which was then tabooed by the profession, and has since become so respectable.

Thackeray considered that he owed his life to Dr. Elliotson.—*Homœopathic World*, London, June 4th, 1898.

